# Comparing Different Filter-Parameter for Pre-Processing of Brain-Stimulation Evoked Motor Potentials

**DOI:** 10.3390/brainsci11091118

**Published:** 2021-08-24

**Authors:** Volker R. Zschorlich, Fengxue Qi, Norbert Wolff

**Affiliations:** 1 Department of Sport Science, University of Rostock, Ulmenstraße 69-House 2, 18057 Rostock, Germany; bewegungswissenschaften@uni-rostock.de; 2 Department Ageing of Individuals and Society, Faculty of Interdisciplinary Research, University of Rostock, 18147 Rostock, Germany; 3 Department of Sport Science, University of Oldenburg, Ammerländer Heerstraße 114-118, 26129 Oldenburg, Germany; 4 Sports, Exercise and Brain Sciences Laboratory, Beijing Sport University, Beijing 100084, China; fengxue.qi@hotmail.com

**Keywords:** brain stimulation, digital filter, MEP, reliability, TMS

## Abstract

Background: Brain stimulation motor-evoked potentials (MEPs) are transient signals and not periodic signals, and thus, they differ significantly in their properties from classical surface electromyograms. Unsuitable pre-processing of MEPs due to inappropriate filter settings leads to distortions. Filtering of extensor carpi radialis MEPs with transient signal characteristics of 20 subjects was examined. The effects of a 1st-order Butterworth high-pass filter (HPF) with different cut-off frequencies 1 Hz, 20 Hz, 40 Hz, and 80 Hz and a 5 Hz Butterworth high-pass filter with degrees 1st, 2nd, 4th, 8th-order are investigated for the filter output. Results: The filtering of the MEPs with an inappropriate filter setting led to distortions on the parameters peak-to-peak amplitudes of the MEP (MEP_pp_) and the absolute integral of the MEP (MEP_area_). The lowest distortions of all of the examined filter parameters were revealed after filtering with the lowest filter order and the lowest cut-off frequency. The 1st-order 1 Hz HPF calculation results in a difference of −0.53% (*p* < 0.001) for the MEPpp and −1.94% (*p* < 0.001) for the MEParea. Significance: Reproducibility is a major concern in science, including brain stimulation research. Only the filtering of the MEPs with appropriate filter settings led to mostly undistorted MEPs.

## 1. Introduction

**Figure 1:**
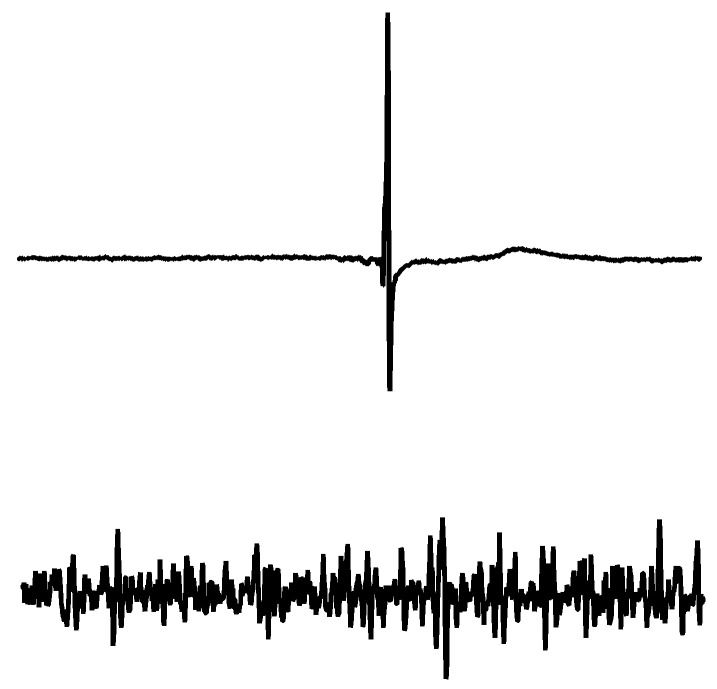
A measured raw data set of an MEP (upper) and interference EMG (lower) shows the different properties of a transient signal and a continuous signal. The upper graph shows a compound muscle action potential (CMAP) curve induced by a transcranial magnetic stimulation (TMS). The lower surface EMG recording shows the stochastic properties of an interference EMG during a slight voluntary contraction. The time scale shows a signal duration of one second and an amplitude of ±1.75 mV. Signals belongs to the same subject with the same electrode application on the extensor carpi radialis (ECR).

The aim of digital filtering is the removal of noise and artefacts from signals. The recording of motor-evoked potentials (MEPs) is not always possible, without different kinds of noise contamination, such as a power line hum [1,2,3], movement artefacts [4], stimulation artefacts, electrocardiographic (ECG) artefacts [5,6] and contamination of baseline wander [4,7,8]. A prerequisite for calculating the MEPs is, however, that the signal of the muscle response is free of noise and artefacts. In the early years of electromyographic recording, analogue filtering was a standard procedure in surface electromyography (sEMG) measurements. Before digital signal processing, an analogue filter was the solution for getting suitable results for a further calculation of the signals online. With the availability of digital calculators, a digital filter [9] was thereafter routinely used for processing the sEMG signals. Different types of digital filters and different parameter layouts have been used extensively in sEMG signal processing, but the use of filters in bio-signal analysis is not always as simple as it may look [4,10]. Distortions of electroencephalography (EEG) signals after filtering have been discussed recently [11,12,13,14], but there is no recommendation for filtering MEPs. MEPs are different from interference electromyograms (Figure 1). 

**Figure 2:**
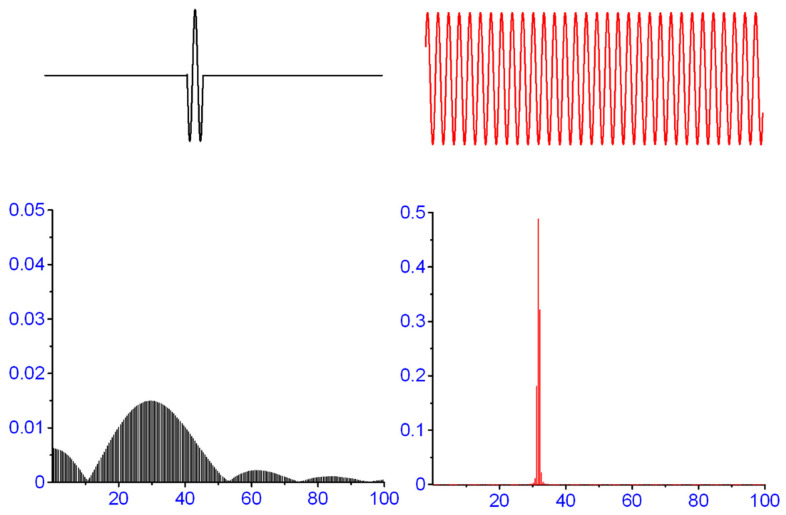
A simulated data set of an MEP (three half-cycled sine wave on the left) and a continuous sine wave (right) with the same periodicity. The different degrees of signal representation resulted in a different frequency spectra quality. The corresponding spectra are arranged below. Note: the amplitude scale (arbitrary units) on the right spectrum is 10 times higher, while the spectrum of the left shows important spreading of the original frequency (arbitrary units) into the adjoining frequency bins. The two signals in the time domain represented, on the one hand, a signal with a transient property (impulse) and on the other, a signal with a clearly periodic property.

The control process of an MEP signal generation is not the same as it is for an interference EMG. The resulting MEP-signal of a stimulation procedure is, in terms of signal analysis [15], a *transient signal* and is, therefore, not a *periodic signal* like a sine-wave, and further, it is also not a stochastic-ergodic process. This is the essential and serious difference between an MEP and an interference EMG and this must be taken into account. An MEP is a transient signal that is elicited by transcranial magnetic stimulation (TMS). The MEPs consist of the activity of many motor units (MUs) that are triggered nearly at the same time, with the same stimulus and every single activated MU adds to and interferes with a compound muscle action potential (CMAP). This process results in a completely different data characteristic. System analysis distinguishes between signal types, periodic signals and transient signals. This is crucial for all signal processing procedures, including filtering (see Figure 2).

Numerous amount of studies have used these same filter parameters with band-pass filtering (5–500 Hz) which have been proposed for a surface EMG from the International Society of Electrophysiology and Kinesiology (ISEK) [16]. Other recommendations came from the Surface EMG for non-invasive Assessment of Muscles (SENIAM) Proceedings, which have suggested a high-pass filter (HPF) with a cut-off frequency in the range of 5 Hz up to 20 Hz [17,18].

In addition to the general recommendations for handling surface-myograms, International Federation of Clinical Neuroscience (IFCN) consensus groups [19,20] have made very detailed recommendations for the measurement of MEPs. The recommendations of the consensus groups are based very closely on the recommendations of the ISEK [16] and SENIAN [18] groups. The peculiarities of the signal properties of an MEP are not specifically taken into account. This in turn has the consequence that the calculation of the parameters of the MEP is exposed to considerable distortions for specific filter settings.

These approaches for interference EMGs cannot be validated for the use of CMAP filtering including TMS-evoked MEP. The main reason is that an MEP is a transient signal and that transient non-periodic signals do not meet the criterion of ergodicity. Ergodicity means that the statistical properties of a measured signal remain constant over the entire measurement period. This is not the case with an MEP and the violation of the ergodicity in a measurement process means that considerable distortions must be expected in the further processing of the signals. Thus, the calculation methods of filtering MEPs leads to more or less strong distortions. Consequently, the purpose of this study was to examine the effects of different cut-off frequencies and filter-orders on signal properties of the MEPs. In this paper we describe the distortion effects of filtering the MEPs, together with an identification of their consequences quantitatively. The processing of stochastic-ergodic signals (interference EMG) remains nearly unaffected by the distortion problems we dealt in this study.

## 2. Considerations for Signal Processing

The sEMG signal as an interference electromyogram is a stochastic signal. Therefore, any operation on this signal is a statistical operation. It is essential for many further calculation procedures of the time series that the signal must meet the assumption of stationarity. Further, the ergodic theorem must be taken into account in the signal representation. In general, the recording duration of a slow process may be critical and the recording must be long enough for an adequate representation of the underlying process. Furthermore, the analogue signal must be represented with sufficient accuracy, both in the areas of the amplitude-resolution and of the time-resolution. This means that the signal is measured with a sufficient sampling rate and that the appropriate sampling rate depends not only on the frequency content but also on the propagation processes in the investigated system. For details on signal processing, we refer to textbooks [15,21].

### 2.1. Signal Stationarity 

There are basically two types of data sets: *deterministic* signals and *stochastic* signals. Deterministic signals are characterized by the fact that they can be described, in principle, in the form of a mathematical function. They are assigned to a function value of x, at any time of t. The unique property of stochastic signals is that they are unpredictable, in the sense that one cannot calculate a value x(t) of the signal function for a certain time t; but the expectation value of the function is given. These forms of signals are called stochastic. Stochastic signals are usually described by using statistical methods. A stochastic process is stationary, if the parameter values of different time periods t_1_, t_2_ and t_3_ with the same statistical properties exist; this means that the same *expectation values* and the same *variances* for the different composite functions can be found. Depending upon what is due to the unsteady behavior of the stochastic process (e.g., artifacts, amplifier drift and thermal noise), the signal must be filtered or discarded. In practice, the cause of non-stationary data sets often lies in a time variance of the signal-generating systems, as is typically the case with biological systems [22].

### 2.2. Ergodic Theorem 

Another basic assumption for the investigation of stochastic signals is the validity of the ergodic theorem. The ergodic theorem states the following: the measured signal of a stochastic-ergodic process must have constant statistical properties over the entire measurement period. It is crucial that every ergodic process is always stationary. Transient non-periodic signals as an MEP do not meet the criterion of ergodicity. Thus, one cannot directly apply the calculation methods of filtering without strong distortions, as in the case of filtering an MEP.

**Figure 3:**
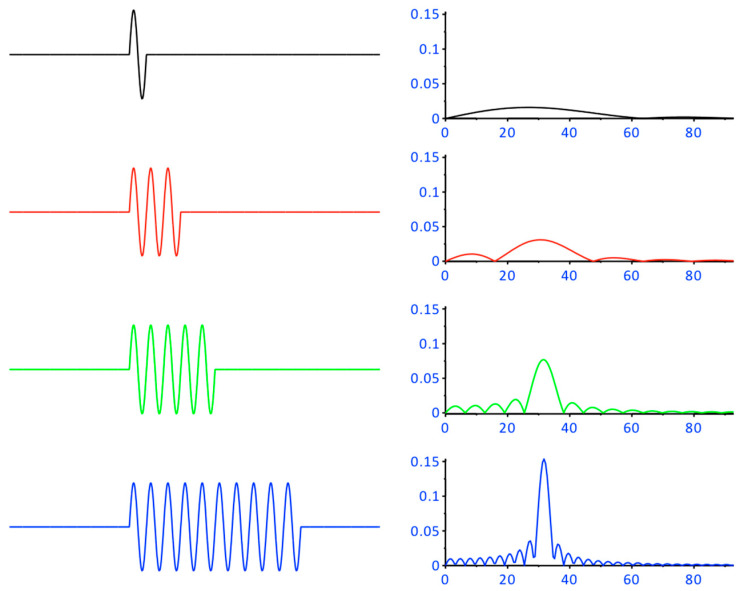
On the left side of the figure, pure sinusoidal oscillations are presented which differ in the number of oscillations but are of the same frequency. The calculated spectra of the sinusoidal oscillations (same frequency!) are shown on the right-hand side (arbitrary units). The upper signal with one full sine wave comes very close to an MEP. The weak representation of this upper signal in the frequency domain on the right is easy to recognize. The better the representation of the signal in the time domain, so does the representation in the frequency domain.

The violation of the ergodic theorem is appropriately severe. The significance of the violation of the ergodic theorem can simply be demonstrated in a simulated case of a transformation from the time domain to the frequency domain. For this purpose, transformations have been carried out which transforms a single sine wave and produce a frequency response with extensive leakage. With an increasingly better representation of the signal in the time domain (3, 5 and 10 sine waves), the representation in the frequency domain also increases which is illustrated in Figure 3.

## 3. Methods

### 3.1. Subjects

Twenty healthy subjects (13 male/7 female, age 27.7 ± 8.7 years) participated in this study. The volunteers were familiarized with the stimulation procedure before. The study was conducted according to ethical considerations [23] as required by international standards. Ethical approval for the stimulation experiment was given by the ethics committee of the University Rostock—Germany. Participants gave their written consent to participate in the investigation.

### 3.2. Study Design

**Figure 4:**
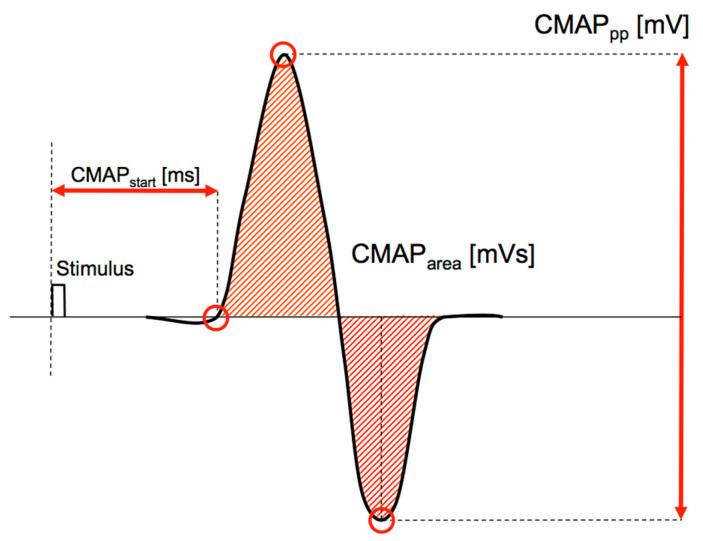
A simulated biphasic waveform of an MEP. The different parameters of the signal represents the main issues defining the influence on cortico-spinal excitability. The initial negative phase of the MEP (left side) is not considered in this calculation.

**Figure 5:**
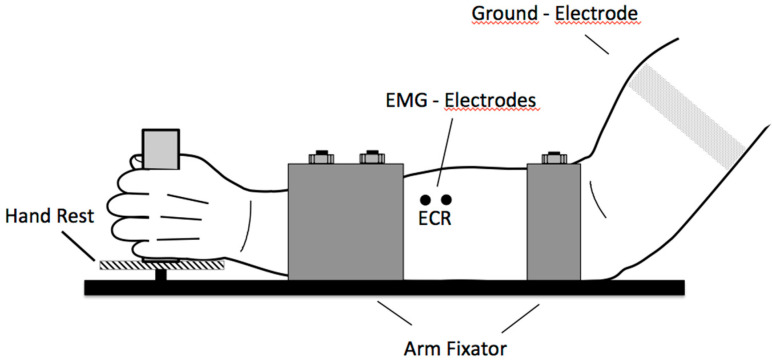

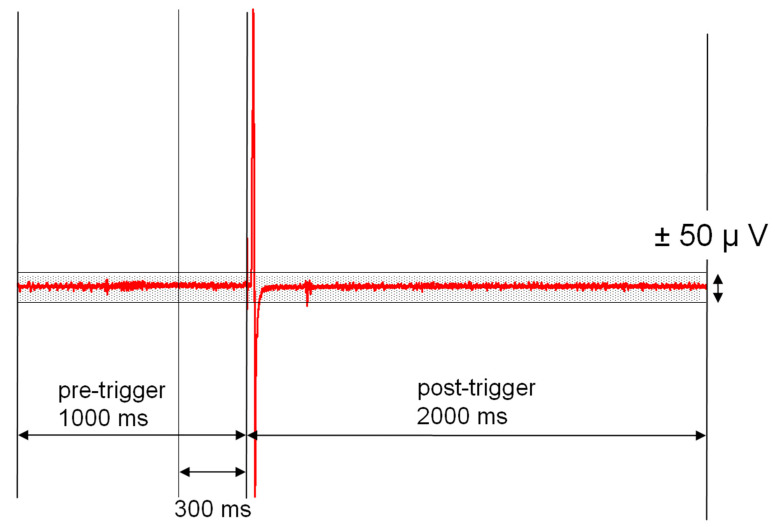
The MEP used for the evaluation of the different digital Butterworth filter-parameter were recorded in a completely relaxed state supported with a hand rest and a lower arm fixator. The recorded time window was of 3000 ms duration. The MEP raw data (lower part) shows a single measurement of a typical data set with 1000 ms pre-trigger. The window for the elimination of background activity was 300 ms before trigger with an amplitude of ±50 µV.

The effects of filtering MEP signals with clear transient characteristics were tested quantitatively in this study. The MEPs of 20 subjects were investigated. The EMGs examined were nearly uncontaminated by noise or hum and had no offset. Maximum care was taken to ensure that the EMG signal was only minimally contaminated. Movement artifacts were ruled out by slight fixation of the test subject. Power line hum was largely reduced due to shielding and power supply to the measuring apparatus by DC. The examination of MEPs serves for the quantitative estimation of the corticospinal excitability (Figure 4). Under optimal stimulation conditions, biphasic responses to the TMS can be measured. This means that the excited motor units are elicited at almost the same time. In this case, the evoked potentials, by means of the TMS, can be precisely determined by peak-to-peak amplitude values. All of the calculated MEPs in this experiment had a biphasic property (see Figure 5). From these MEPs, the peak-to-peak amplitude (MEP_pp_), and the absolute area (MEP_area_) were calculated in a time-window of 100 ms and the runtime of the signal (MEP_start_), after the triggering of the TMS were calculated. MEP_start_ was calculated backwards in the time domain from the first positive peak of the MEP signal until crossing the zero line. The first positive value after this baseline crossing was detected as the MEP_start_ parameter. MEP_pp_ and MEP_area_ were used to determine the corticospinal excitability and MEP_start_ was used to determine the latency—the time between the stimulus and the response. The time parameter also showed the sensitivity of the MEPs to filter procedures in the determination of the cortical silent periods (CSP) or similar runtime relevant procedures. We investigate the effects of filtering with different filter parameters on the signal properties and test them for statistically significant differences compared to the raw-signal. We chose a 1st-order high-pass Butterworth filter, with different cut-off frequencies of 1 Hz, 20 Hz, 40 Hz, and 80 Hz for this study. We also varied the order of a 5 Hz high-pass Butterworth filter with degrees of the 1st, 2nd, 4th and 8th-order. The selected cut-off frequencies and the filter settings that were applied in this study have reflected in parts the recommendations of a sEMG processing [18] and were based, furthermore, on previous meta-analysis studies using measurements of the MEPs [24,25,26,27].

### 3.3. MEP Release and Recording

By means of a pulsed magnetic stimulation over the right primary motor cortex (M1) [28,29], an EMG response was triggered in the left extensor carpi radialis (ECR) muscle (Figure 5). This muscle response was initially achieved through the triggering of an action potential in the associated motor neurons in the M1. Single-pulse magnetic stimulation was carried out using a Magpro 30+ stimulator with the Mag-Option (MagVenture (formerly Medtronic), Skovlunde Denmark) and a figure-of-eight coil type MC-B70 was used. The stimulator generated biphasic symmetric pulses with a duration of 290 µs. The coil was placed over the right primary motor cortex where the lowest motor threshold (response > 100 µV) could be found in 3 of 5 responses for the recorded ECR. 

Subjects were sitting upright on a chair with the left arm rested in a comfortable abduction of the upper arm and the forearm slightly fixed in a relaxed position. The head was stabilized with a forehead-chin-rest to reduce head-to-coil movements. The left arm was bent at the elbow joint. The participants were instructed to focus on a cross on a video screen (distance 110 cm).

The stimulation site was about one to two cm behind the vertex. The coil position was kept constant with the help of two adjustable arms (Manfrotto, Feltre, Italy) during the experiment. The magnetic gradient for all subjects lay between 40–70 A/µs (this corresponds to 27–48% of the maximum stimulator output) and resulted in MEPs in a range between ± 0.70 mV and ± 2.85 mV. Only MEPs were included, whenever surface EMG amplitude was lower than ± 50 µV in a time window 300 ms before the onset of an MEP. All higher background EMG activity indicates a voluntary muscular activation and has a strong impact on MEP amplitudes [28].

The TMS elicited MEPs were recorded with an AC differential amplifier (biovision, Wehrheim, Germany) with an input resistance of 10 gΩ and a bandwidth of 0.2 Hz to 1000 Hz (3 dB/octave). The gain was chosen with a factor of 1000. We would like to point out that some manufacturers of TMS equipment also offer EMG amplifiers. However, these amplifiers often show bandwidths with lower cut-off frequencies of 10 Hz or 20 Hz. This amplifier design may result in considerable distortion of the EMG signal and only a few manufacturers offer amplifiers with an adequate bandwidth.

In this study, MEPs were registered with two Ag/AgCl cup electrodes (Hellige baby-electrodes; GE Medical Systems, Milwaukee, WI, USA) with an electrode surface area of 3 mm^2^ and were placed at a distance of 1 cm longitudinally over the belly of the ECR near to the innervation zone. The reference electrode was placed at the acromion. The skin preparation before electrode application was carried out with alcohol and the hairs were removed. Skin abrasion was performed to reduce surface keratin layers and reduce skin capacitance. The skin impedance at 30 Hz was always lower than Z = 5 kΩ. 

For conductance, an electrode gel (Parker Laboratories, Fairfield, CT, USA) was used. Electrodes and twisted cables were fixed with self-adhesive tape on the skin. We recorded our MEP individually with a pre-trigger of 1000 ms and a measurement period of 3000 ms. Signals were recorded with a DAQ-Card 6024 (National Instruments, Austin, TX, USA) at 12-bit resolution and a sampling rate of 10,000/s, using the DIAdem 8.1 (National Instruments) signal processing program. The DAQ-Card 6024 samples the data with a referenced single-ended (RSE) configuration. It should be mentioned that only measured EMG raw-data can be filtered in a pre-processing act. Averaged data from several trials, an ensemble of MEPs, cannot be filtered post-hoc. The EMG was calculated with a Butterworth HPF with different cut-off frequencies, different filter orders and different transfer properties. The calculation of the filter process was carried out in the time domain with the signal analysis module of DIAdem.

## 4. Statistics

In this study, the average parameter values of the respective filtered data were compared with the calculated parameters of the unfiltered raw data, when considering the most important parameters of MEP_area_, MEP_pp,_ and MEP_start_. Repeated measures of the one-way analysis of variance (ANOVA) were used to compare the effects of the different filter settings on the raw data. The homogeneity of variances was tested by Shapiro-Wilk-Test. The assumption of sphericity was checked with the Mauchly test. A Greenhouse-Geisser correction was carried out if necessary.

A post-hoc analysis with Bonferroni correction was used to confirm the differences between the raw data and the filtered data. The effect size (partial eta square, η_p_^2^) was reported. The data were analyzed by using SPSS Version 22.0 for Windows (SPSS Inc., Chicago, IL, USA). A *p*-value of <0.05 was considered significant.

## 5. Results

### 5.1. Variation of the Cut-Off Frequency

**Figure 6:**
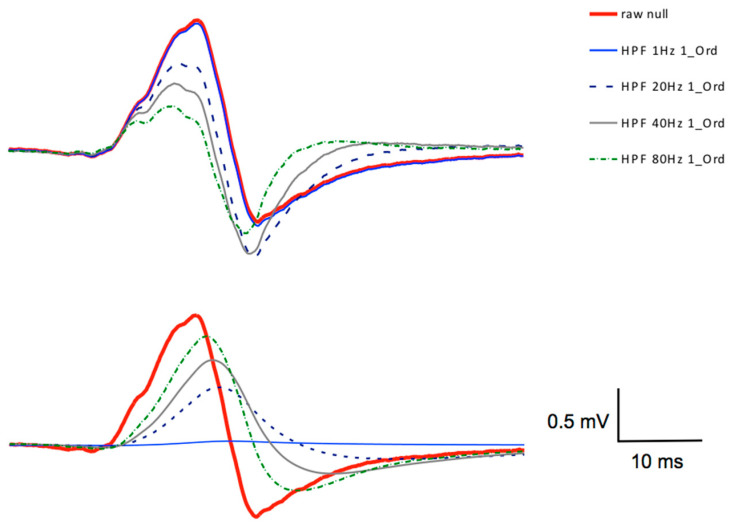
The single unfiltered MEP raw signal from a representative participant is shown as a continuous bold red line. The other signals (on the upper graph) were filtered with a 1st-order Butterworth HPF with different cut-off frequencies (1 Hz, 20 Hz, 40 Hz, and 80 Hz). The time window in this graph was 100 ms in duration. The graph shows the MEP signal 1 ms after TMS trigger. The lower graph shows the areas of deviation of the different filtered signals to the unfiltered MEP.

The variations of the cut-off frequencies of the 1^st^-order Butterworth high-pass filter demonstrated marked differences in several MEP parameters of the extensor carpi radialis, especially with the higher cut-off frequencies. The differences (Δ%) of the evaluated MEP parameters are shown in Table 1. The Shapiro-Wilk-Test meet the assumption of homogeneity of variances in all cases. The Mauchly test did not meets the assumption of sphericity, therefore a Greenhouse-Geisser correction was carried out. The statistical analyses revealed cut-off frequency-dependent significant differences in the parameters of MEP_area_ (*F*_4,76_ = 157.82, *p* ≤ 0.001, η_p_^2^ = 0.893) and MEP_pp_ (*F*_4,76_ = 201.14, *p* ≤ 0.001, η_p_^2^ = 0.914). The MEP_start_ parameter showed no significant differences in all of the cut-off frequency variations (*F*_4,76_ = 2.07, *p* ≤ 0.092, η_p_^2^ = 0.098). The results of the filtering process with different cut-off frequencies showed distortions of the original MEP signal, as seen in Figure 6. 

The results of the 1st-order filter with 1 Hz HPF showed only a small distortion of the MEP amplitude. The 20 Hz filter resulted in a decline of the amplitudes. The 40 Hz and the 80 Hz filtering resulted in a further strong amplitude reduction and also in an oscillation (a swinging tail of the MEP, not visible in the short time-window of Figure 6).

For the MEP_area_ parameter, the results showed significant differences in the filtered data for all of the cut-off frequencies in the 1 Hz (*p* ≤ 0.001), 20 Hz (*p* ≤ 0.001), 40 Hz (*p* ≤ 0.001), and 80 Hz (*p* ≤ 0.001) 1^st^-order Butterworth HPF, compared to the raw data. The 1 Hz high-pass filter in this case shows the best results. 

For the MEP_pp_ parameter, the post-hoc analysis showed significant differences in the 1 Hz, 40 Hz and 80 Hz 1st-order Butterworth HPF in comparison with the raw data (all values of *p* ≤ 0.001), but did not significantly differs for the 20 Hz 1st-order Butterworth HPF data (*p* = 0.052). The time parameter MEP_start_ was robust against the filtering and it showed no statistical differences in the case of a simple HPF. 

The amount of distortions of the MEPs after filtering became clearer after the visualization of the deviations (Figure 6, lower part). The percentage deviations of the filtered signals showed strong differences for MEP_pp_ and MEP_area_ in nearly all of the filter settings, as compared with the raw data. The distortions of the 20 Hz, 40 Hz and 80 Hz filtering resulted in remarkable differences, which ensued from a substantial amplitude reductions in all of the cases.

### 5.2. Variation of the Filter-Order

The variations of the order of the Butterworth high-pass filter, with a cut-off at 5 Hz, demonstrated that not only different cut-off frequencies influenced the outcomes of the filtering procedures, but also the orders of the filter transfer-functions had a remarkable impact. The 5 Hz Butterworth HPF was varied with a 1st-order, a 2nd-order, a 4th-order and a 8th-order transfer characteristics for comparison. The statistical analyses of the different filter-orders revealed significant order-dependent differences in the parameters of MEP_area_ (*F*_4,76_ = 14.34, *p* ≤ 0.001, η_p_^2^ = 0.430) and MEP_pp_ (*F*_4,76_ = 30.67, *p* ≤ 0.001, η_p_^2^ = 0.885), but the MEP_start_ parameter showed no significant differences (*F*_4,76_ = 1.66, *p* = 0.20, η_p_^2^ = 0.294).

**Figure 7:**
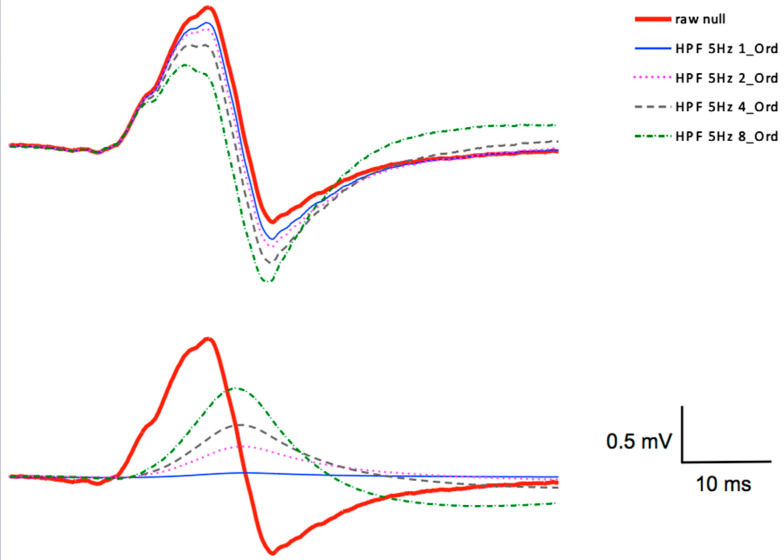
The surface electromyogram of the unfiltered MEP is shown as a continuous red line from the same subject as before. The signal was filtered with a 5 Hz Butterworth HPF with different orders, a filter with 1st-order, with 2nd-order, with 4th-order and with 8th-order was chosen for comparison. The time window in this graph was 100 ms in duration. The 1st-order filter shows the smallest difference compared to the raw signal. The lower graph shows the areas of deviation of the different filtered signals to the unfiltered MEP.

For the MEP_area_ parameter, the post-hoc test showed significant differences between the raw data and the filtered data for the 8th-order filter (*p* ≤ 0.01). For the MEP_pp_ parameter, the results revealed significant differences for the 5 Hz 1st-order Butterworth HPF (*p* ≤ 0.01), the 5 Hz 2nd-order Butterworth HPF (*p* ≤ 0.001) and the 4th-order Butterworth HPF (*p* ≤ 0.05) as compared to the raw data, but in contrast the 5 Hz 8th-order Butterworth HPF was robust again and showed no significant differences to the raw data. The time parameter MEP_start_ showed no statistically significant differences between the raw data and the filtered data in all cases of the different filter-order with the 5 Hz HPF (Figure 7).

The study further considered the percentage deviations (Δ%) of the parameters of MEP_area_, MEP_pp_, and MEP_start_ for the evaluation of the measured MEPs when using all of the calculated filters. The absolute differences are shown in Table 2 as percentage deviations (Δ%). It can already be deduced from the deviations of the physical mean values of the filtered data, when compared to the raw data (Table 1), that the direction of the deviation was not linear, when increasing the cut-off frequencies or by increasing the filter-order. The most relevant parameters of MEP_area_ and MEP_pp_ showed already statistically significant differences at a filter setting of 1 Hz HPF 1st-order. Overall, one can say that the different filter settings, demonstrated significant differences, which indicated that the filtering procedures resulted in non-linear related distortions and not simply by displaying a bias.

## 6. Discussion

The purpose of this study was to show that a careful choice of the filter types and the filter-parameters in the removal of artefacts is essential. Reliability is a major objective in science, including brain stimulation research. The correct processing of physiological signals was an important issue [30,31,32] and the unsuitable pre-processing, due to inappropriate filter settings, may be undermining the reproducibility of the results (MEP_pp_, MEP_area,_ and MEP_start_) in non-invasive brain stimulation experiments. The present study demonstrated that muscle responses to a stimulation cannot be carelessly processed, by using the same filter types and filter parameters that are used for interference surface EMG.

The muscular responses of the examined extensor carpi radialis muscle to the stimulation procedures resulted predominantly in a CMAP, likewise with transcranial magnetic stimulation. The resultant MEP was a transient signal (impulse). The TMS or the other stimulation procedures, like the tendon reflex (TR), the Hoffmann-reflex (HR), the transcranial electrical stimulation (TES), the cervico-medullary motor evoked potentials (CMEPs), as well as the field potentials, also resulted in an impulse-like signal shape. By means of a pulsed magnetic stimulation over the primary motor cortex (M1), an EMG response was triggered in the ECR. This muscle response was initially achieved through the triggering of an action potential in the associated motor neurons in the M1. The axons of these upper motor neurons facilitated the associated spinal α-motor neurons. The α-motor neurons conducted the generated action potential to the neuromuscular junction, thus triggering a twitch contraction in the muscle. This short muscle activity was measured by a sEMG. The signal characteristics were transient.

One of the central questions in the context of processing motor evoked potentials is: what is the optimal filter for calculating the characteristical parameters of a transient MEP? The underlying hypothesis was the following: One could safely assume that the application of the pulsed magnetic fields to the motor cortex would result in a signal with dominant transient signal characteristics of the motor unit potential. A validation of the hypothesis, as given in the present paper, has shown the requirement for cautious filter calculations. When taking care of this assumption (transient signal characteristic), it opens up the possibility of obtaining results with a smaller deviation to the raw data. A description of the methods of digital signal analysis can be found in textbooks [15,33]. High-pass filters are typically applied for removing movement artefacts and the baseline wander [7]. 

The experimental variations of the Butterworth HPF were different filter-orders (1st, 2nd, 4th, 8th-order) and different cut-off frequencies (1 Hz, 20 Hz, 40 Hz, and 80 Hz) and they showed the following results: if the cut-off frequency was set exceedingly high for the HPF, then the maximum amplitudes were reduced. This distortion also effects the MEP_area_ parameter. The MEP_pp_ parameter is not equally affected of filtering because the attenuations of the positive amplitude of the signal are compensated to some extent by increasing the negative signal components simultaneously. Thus, the MEP_pp_ parameter was not only relatively robust against lacking stationarity of the mean (expectation value = 0), but also due to a large approximation of the stop-band in the frequency ranges of the effective fraction of the signal. The effects of filtering, with low cut-off frequencies, were substantially more moderate in the HPF, when compared to the higher cut-off frequencies, both in terms of the parameters and the absolute deviations of the waveforms of the MEP. 

Even a conservative filter design (HPF 1 Hz 1st-order) showed statistically significant deviations for the evaluation of the MEPs in the relevant parameters of MEP_area_ with −1.94% and MEP_pp_ with −0.53%. The non-linear transfer behavior and the development of the distortions, owing to less moderate filter designs (higher orders, higher cut-off frequencies), sometimes did not result in significant differences in the responses, when considering the miscellaneous parameters. In some cases, the statistically non-significant results of the ANOVA with repeated measurements, concealed the fact that the non-linear distortions represented relevant deviations in the curve characteristics and that the other important parameters underwent considerable deviations due to the filtering. 

At this point, we would like to point out that some manufacturers of EMG equipment offer models of EMG amplifiers which show bandwidths with lower cutoff frequencies in the range between 10 Hz and 20 Hz. This amplifier design may result in considerable distortion of the MEP signal. Other manufacturers (the list is not exhaustive) offer amplifiers with an adequate bandwidth. These companies have EMG amplifier models in their portfolio that show considerably larger bandwidths and are therefore much more suitable for the acquisition of MEPs. These amplifiers work at factory with lower band limits between 0.05 Hz and 1 Hz and with upper band limits between 10 kHz and 200 kHz. 

Another problem that is associated with the filtering of signals is the wide variety of filtering algorithms that are available in the signal analysis software packages. There are algorithms that perform filter calculations in the time domain (DIAdem and LabVIEW—National Instruments), but there are also software packages in which the filtering optionally takes place in the frequency domain (DASYLab—National Instruments). Efforts to find out which algorithms are actually calculated failed for a variety of reasons. But for a comparison of the measurements, it is indispensable that one works with the same algorithms. In a crash analysis for passenger cars, therefore, a standard (both SAE and ISO) for the low-pass filtering of the measured data has prevailed [34].

One limitation of our study is that we studied nearly uncontaminated signals. This in turn means that we cannot make any statements about the benefit in the area of the signal-to-noise ratio. However, unmasked by artefacts, we can show the distortions caused by the filtering more precisely. This study was also limited in that it only evaluated one filter-type. We restricted our observation to the behavior of the very common Butterworth filter, as this filter is very suitable in its transfer characteristics for time-domain post-processing (no ripples in the pass-band and stop-band). Filtering allows a considerable improvement in the signal-to-noise ratio in principal. This means that, under certain conditions, the filtering of noise-contaminated signals is an essential requirement in order to analyze MEPs quantitatively. 

A very recently published study [35] investigated the problem of filtering MEPs with a view to reducing the signal-to-noise ratio. Here, too, it could be shown that the filters with the lowest cutoff frequency (10 Hz and 20 Hz in this case) showed the lowest amplitude distortion and, moreover, they were able to show that better signal-to-noise ratio could be achieved for these filter parameters as well.

In principle, it is possible, even after filtering the MEPs, that changes in kind of neuroplasticity can be detected, but if the filtering is unfavorable, this can lead to significantly higher variances in the data. We would also like to point out that we only measured and filtered MEPs on the ECR and did not generalize the results to other muscles. 

In order to generate the lowest possible distortion of the MEPs, we recommend that all possible precautions must be taken on the hardware side to ensure that the MEPs are not contaminated. This means that the power line noise must be prevented by careful electrode application including skin preparation, possibly by a Faraday cage or other shielding. The power circuits can also be decoupled, if possible with a direct current battery supply. If all these precautions are taken, one can easily use filters with the lowest order (HPF 1st-order) and filters with low cut-off frequencies (HPF cut-off frequency 1 Hz) in order to obtain the lowest possible distortion of the original signal which generates fewer variances in the data.

## 7. Conclusions

The results of the study demonstrated that the TMS evoked MEPs of the extensor carpi radialis muscle showed a sensitivity for filtering procedures. The sensitivity of the filtering process to distortions of the MEPs resulted from the fundamentally non-periodic properties of a CMAP. On the basis of these properties of the MEP, the following statements can be made for filtering transient MEP signals: (1) The filtering of an MEP is not the same as filtering the quasi-periodic interference surface electromyogram. The signal properties are tremendously different between a transient signal (MEP) and a stochastic signal (interference EMG). All kinds of CMAPs (MEP, CMEP, HR, TR, field potentials) are impulse-like functions, which are not perfectly synthesized by the Fourier series. The filtering of an MEP may result in possible relevant distortions, depending on the filter parameter settings; (2) The interference EMG signal power starts at frequencies higher than 5 Hz [9]. In order to obtain a “weak stationarity” of the measured signal, necessary for the quantitative evaluations of the MEPs, only a high-pass filter with very low cut-off frequencies should be used. High-pass filters higher than 1 Hz cut-off frequency cannot be applied without important distortions of the MEP signal; (3) Unnecessarily high filter-orders (greater than the 1st-order) should be avoided, because they may result in substantial distortions of the signal.

## Figures and Tables

**Table 1:** In response to the different filter settings, the absolute percentage differences (Δ%) of the MEP-parameter between the raw data and the filtered signals are shown in the table. Significant differences in MEP characteristic parameters are presented. *ns =* not significant, * *p* < 0.05, ** *p* < 0.01, *** *p* < 0.001.

Filter	MEP_area_	MEP_pp_	MEP_start_
HPF 1 Hz—1. Ord	*** −1.94	*** −0.53	*ns* 0.80
HPF 20 Hz—1. Ord	*** 17.24	*ns* 3.10	*ns* −3.81
HPF 40 Hz—1. Ord	*** 32.23	*** 15.09	*ns* −3.88
HPF 80 Hz—1. Ord	*** 51.25	*** 35.51	*ns* −7.87
HPF 5 Hz—1. Ord	*ns* −0.50	** −1.46	*ns* −4.26
HPF 5 Hz—2. Ord	*ns* −2.65	** −2.58	*ns* −1.73
HPF 5 Hz—4. Ord	*ns* −1.34	* −3.17	*ns* −1.63
HPF 5 Hz—8. Ord	** −7.40	*ns* −1.10	*ns* −4.19

**Table 2:** MEP parameters of the ECR muscle are presented. Raw data (MEP_raw_) were used as a reference value. The filter parameters vary in the cut-off frequency of the Butterworth HPF were chosen, while the order of the filter was kept constant at 1st-order (High-Pass Filter 1.Ord). MEP parameters (5 Hz High-Pass Filter) were shown against filter properties at different filter orders, while the cut-off frequency of the high-pass was set to 5 Hz. Values are the differences which were averaged over all subjects and presented as the mean ± standard deviation (SD).

		High-Pass	Filter 1.Ord		
Parameter	Raw Data	1 Hz	20 Hz	40 Hz	80 Hz
MEP_start_ (ms)	14.40 ± 2.62	14.29 ± 2.82	14.95 ± 2.73	14.96 ± 2.72	15.54 ± 2.21
MEP_pp_ (mV)	1.80 ± 0.51	1.81 ± 0.52	1.74 ± 0.50	1.52 ± 0.47	1.16 ± 0.38
MEP_area_ (mVs)	10.35 ± 2.70	10.55 ± 2.69	8.56 ± 2.13	7.01 ± 1.75	5.04 ± 1.31
		**5 Hz High-Pass Filter**			
Parameter	Raw Data	1.Order	2.Order	4.Order	8.Order
MEP_start_ (ms)	14.40 ± 2.62	15.02 ± 2.45	14.95 ± 2.73	14.64± 2.78	15.51 ± 2.71
MEP_pp_ (mV)	1.80 ± 0.51	1.82 ± 0.51	1.84 ± 0.51	1.85 ± 0.52	1.82 ± 0.53
MEP_area_ (mVs)	10.35 ± 2.70	10.40 ± 2.58	10.62 ± 2.64	10.49 ± 2.62	11.12 ± 2.76

## Data Availability

The data presented in this study are available on request from the corresponding author.
